# Closing the Automation Gap: Robust AI for Dual-Stain Cervical Cancer Screening Triage

**DOI:** 10.21203/rs.3.rs-5985837/v1

**Published:** 2025-03-04

**Authors:** Bernd Lahrmann, Andreas Keil, Felipe Miranda Ruiz, Megan A. Clarke, Didem Egemen, Kiranjit K. Grewal, Finley P. Grabe, Liam Bartels, Alexandra Krauthoff, Philipp Ströbel, Carolann Risley, Sydney Reaves, Laurie A. Fuller, Walter Kinney, Nancy Poitras, Patricia E. Goldhoff, Betty Suh-Burgmann, Thomas S. Lorey, Nicolas Wentzensen, Niels Grabe

**Affiliations:** 1 Steinbeis Center for Medical Systems Biology, Heidelberg, Germany;; 2 Institute of Pathology, University Medicine Goettingen, Goettingen, Germany;; 3 Hamamatsu Tissue Imaging and Analysis Center TIGA, University Heidelberg;; 4 Heidelberg University, Medical Faculty Heidelberg, Department of Medical Oncology, Heidelberg University Hospital, Heidelberg, Germany; 5 D120/DKFZ, Clinical Cooperation Unit Applied Tumor Immunity, German Cancer Research Center, Heidelberg, Germany; 6 Kaiser Permanente Northern California, CA USA;; 7 University of Mississippi Medical Center, Jackson, MS, USA;; 8 National Cancer Institute, Bethesda, MD, USA.

## Abstract

Dual-stain cytology, using p16 and Ki67, is superior to conventional PAP cytology for triage of HPV-positive test results in cervical cancer screening. Its AI-based evaluation can remove subjectivity, improve performance and facilitate implementation. Using 5,722 dual-stain slides from population-based screening cohorts, we developed and validated Cytoreader-V2. In the SurePath Kaiser Implementation Study, Cytoreader-V2 achieved 87.2%/57.8% (sensitivity/specificity) compared to 89.9/52.6 (manual DS) and 85.8/41.9 (Pap cytology). In the Thin-Prep Biopsy Study, it reached 95.7/44.4 versus 89.4/35.0 (manual DS), and in anal DS cytology slides, 87.0/41.3 compared to 87.0/27.7 (manual). Robustness testing demonstrated significant stability across image transformations. Cytoreader-V2 improves specificity and reproducibility compared to manual dual-stain reading while maintaining high sensitivity. Its adaptability across populations with consistent performance makes it scalable for diverse clinical settings. Cytoreader-V2 can be a transformative tool in global cervical cancer screening as a critical AI applications in digital pathology.

## 1 Introduction

Cervical cancer screening is one of the most globally impactful cancer preventive measures and stands out due to its high degree of automation when primary HPV testing is used, which enables efficient and large-scale implementation but is lacking specificity. Therefore, effective triage and management of HPV-positive women is critical to avoid unnecessary colposcopy referrals and associated harms while maintaining high sensitivity for cervical precancer [6]. Triage with p16/Ki-67 dual-stain (DS) testing has shown high sensitivity and specificity for detection of cervical precancers; however, its currently missing automation is hindering global implementation and adoption of the assay itself, in turn restricting the full potential of HPV-based cancer screeening [5, 7, 8, 9, 10]. Dual-stain (DS) has already been included as an important component in the US Enduring Consensus Guidelines [32] and the World Health Organization (WHO) cervical cancer screening guidelines as a recommended triage method for managing HPV-positive individuals [31]. Compared with PAP cytology, dual-stain requires fewer subsequent colposcopies and detects more, and earlier, cervical intraepithelial neoplasia grade 3 or greater [33]. We developed and evaluated Cytoreader V2 as a well-generalizing AI-classifier for dual-stain detection based on our earlier work [13]. Automation of the dual-stain test has the potential to become an impactful application of artificial intelligence in cancer screening for women and could significantly advance global efforts to reduce cervical cancer mortality, with applicability in low-resource settings.

Although advanced machine learning techniques are gaining in complexity with increasing speed [38] using e.g. foundation models [34] or multimodal AI [35], this complexity hinders rather than promotes the introduction of AI into medical practice as the higher a model’s complexity, the higher it’s number of parameters and the more difficult it will be to validate. Despite numerous publications and methodological advances in AI, clinical adoption of AI systems in diagnostics remains extremely limited. Only 26 digital pathology AI based algorithms currently have IVD approval in the European Economic Area [1] and only 4 have been approved by the FDA in the US [37]. Yet, understandable AI with a solid validation against ground truth should facilitate regulatory approval. In contrast to most artificial intelligence (AI) classifiers aiming to match manual reading accuracy, we evaluate our classifier against histological ground truth in three epidemiological studies. This way, we can directly compare Cytoreader to an objective common “truth” against which also manual DS reading and cytological PAP reading as the current diagnostic practice are compared. Comparing tile-level and patient-level predictions then allows us to show that our AI-architecture was sufficiently complex and efficient for clinical implementation.

The Cytoreader-V1 system [13] utilized single-layer scanning of liquid cytology prepared samples (ThinPrep or SurePath) with Whole Slide Imaging (WSI) Devices (Hamamatsu Nanozoomer) [11, 12]. Compared to V1, we substantially enlarged image training data, using iterative computational training data enrichment. Thereby, we could overcome the previously necessary two different separate classifiers for the different appearance of Thinprep and Surepath slide preparation. Next, we developed a robust two-step prediction method consisting of a base classifier and an test-time augmentation boosted ensemble. Subtle image variations, often unnoticed by humans [20], can impact the accuracy of CNNs, as shown in distinguishing skin lesions [19] which also can be surprisingly sensitive to minor image transformations, see [18]. Different techniques like Monte Carlo Dropout [24], Test time augmentation (TTA) [22, 29], Deep ensembles [25] have been evaluated [23]. In Cytoreader-V2 we combined TTA with ensemble machine learning techniques. In the subsequent results section, we evaluated accuracy and robustness of Cytoreader V2 to perform this dual-stain detection with clinical grade quality.

## 2 Results

### 2.1 Classification workflow

**Figure 1:**
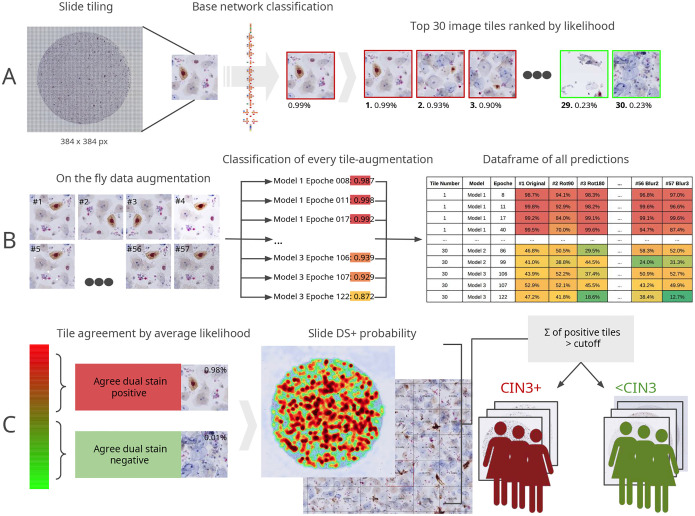
Workflow for image classification. (A) Tile segmentation and initial classification: Whole-slide images are partitioned into tiles of dimensions 384×384 pixels. These tiles are initially evaluated by a base neural network. The top 30 tiles, ranked by their likelihood scores, are selected for subsequent in-depth analysis. (B) Data augmentation and ensemble building: Each of the 30 tiles undergoes 57 distinct data augmentations. These augmented tiles are then independently classified by all models in an ensemble. The collective predictions from these models are combined into a single dataframe, serving as the basis for subsequent statistical analysis. (C) Tile-level and slide-level classification: The average likelihood score, calculated across all augmentation-epoch combinations for a tile, is employed to categorize it as either ‘dual stain positive’ or ‘dual stain negative’. On the slide level, these categorizations are visualized through a heat map. Subsequently, a composite slide score is computed, derived from the proportion of positive tiles exceeding a predetermined cutoff.

The classification workflow first performs exhaustive tiling of the whole slide image and uses a convolutional neural network (CNN) **base classifier** to determine a candidate set of fixed size of the most likely precancerous or cancer containing dual-stain tiles (Figure 1 A). On SurePath slides, 8,000–10,000 tiles, and on ThinPrep slides, 18,000–30,000 tiles are generated and evaluated. Each candidate tile is then subjected to 57 test time data augmentations and each of those augmented tiles is then subjected to a **CNN ensemble** consisting of 30 CNN models, creating a matrix of 1,710 classification likelihoods (augmentations × ensemble models) (Figure 1 B). This matrix is aggregated into a final likelihood for each original dual-stain tile by averaging, while excluding tiles whose likelihood data frame shows a too high standard deviation (STDMAX) and whose classification would be ambiguous. All tiles excluded by STDMAX thresholding still get a reported average likelihood and could be considered e.g. in a potential semi-assisted use of Cytoreader. But to evaluate automatic reading as in this publication, such tiles are just not rated sufficiently positive to call a case dual-stain as positive. Generally, the base classifier exhibits high sensitivity to avoid missing potential precancer events, while the ensemble serves as a subsequent specificity filter. It is important to understand that although the base classifier rates every single tile on the whole slide, here it is used only to generate a fixed number of candidate tiles. For a better understanding of its performance in rating single tiles, Table 2 shows its sensitivity and specificity. Determining the top 30 most likely dual-stain tiles is realizing a filter allowing the following ensembles to perform a fine-grained quantitatively improved judgement as single tiles might be decisive for a positive case. Our approach combines the power of ensemble learning with test-time image augmentation to deliver high-performance image classification for medical diagnostics. By considering the variability of the predictions and reducing the impact of any outlier predictions, it is ensured that the final prediction for each image is not overly influenced by any single model or set of weights.

This workflow creates multiple filter or selection parameters which are described subsequently. First, the size of the candidate set needs to be determined (CANDSIZE). Then, STDMAX, the allowable standard deviation, needs to be defined. We explore in the following the standard deviation limits STD 10, 15, or 20. This approach is known as uncertainty thresholding [27]. Tiles having a data frame with a low enough standard deviation and are thus determinable then are required to additionally have a high enough average likelihood, creating the “LIKELIHOOD THRESHOLD LH” (or short: LIKELIHOOD). Averages exceeding this threshold create “POSITIVE TILES” with cellular “DUAL-STAIN EVENTS”. Finally, the minimum number of positive tiles required to call a case “DUAL-STAIN POSITIVE” we define as the “CUTOFF”. For example 1,2, or 3 positive tiles could be required to constitute a case as dual-stain positive. In the following, those 4 interdependent, mutually linked parameters (CANDSIZE, STDMAX, LH, CUTOFF) were studied and their final selection was determined for robust, sensitive and specific dual-stain classifications.

### 2.2 Classifier development and internal validation

#### Training on tile level and model confirmation on slide level

**Figure 2:**
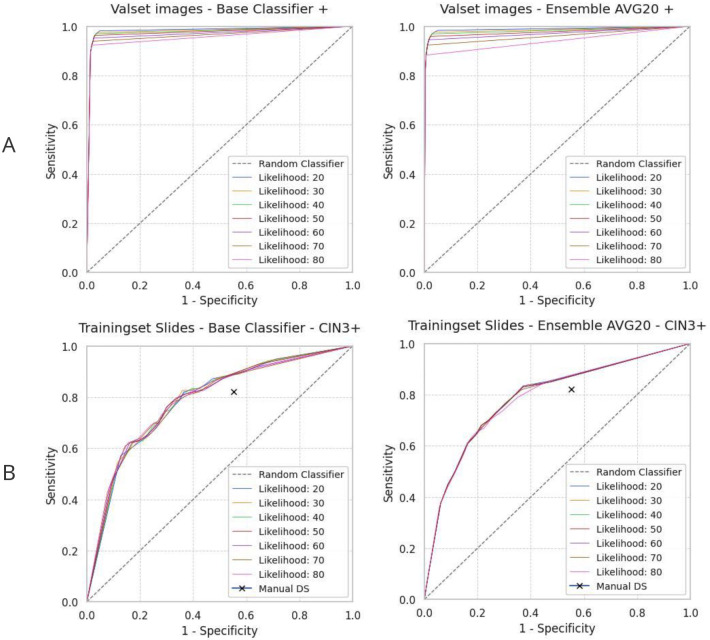
Internal validation performance. (A) Tile-level ROC curve showing very high accuracy (97.2%) and area-under-curve (97.6%) for the base and ensemble classifiers on the 20% validation set showing that model architecture is sufficiently complex and does not require further complexity. (B) Slide-level training set performance. ROC curves for the training slide set for detecting CIN3+ (n = 2423), comparison of manual and automated DS classification. The area under the curves generally exceeds manual review performance, with manual DS performance indicated by a black cross (cutoff of one cell per slide for DS positivity). The stark difference between tile and slide level performance (upper row A vs. lower row B) shows that patient classification cannot further be significantly improved by selecting a more complex AI model architecture, but is instead limited by the realistic ground truth based diagnostic study design and properties of the patient cohort.

For characterizing tile classifier training performance, we analyzed classification accuracy on 11,086 tiles of the original candidate image tile set not used for training. The base and ensemble classifier both show very high accuracy (97.2%) and area-under-curve (AUC) of (97.6%) at 50% likelihood (Figure 2-A). So, tile classification appears nearly perfect when analyzing this independent candidate set. While training happened on the tile level, selection of the final model was confirmed on the slide level in an internal validation step. The primary endpoint for all dual-stain cervical cancer screening studies was CIN3 or greater (CIN3+). Receiver operator characteristics curve analysis is shown for the number of DS-positive cells (cutoffs) for different likelihood thresholds against the primary endpoints. A slide was classified as dual stain positive if there are more than *c* image tiles (cutoff) which have a likelihood higher than *l*. Sensitivity and specificity for manual DS evaluation were plotted on the receiver operator characteristics (ROC) curve for comparison. Figure 2-B shows the ROC curves for the training set slides (n =2423). Compared to tile classification, whole sample prediction showed to be much more challenging as it is performed against ground truth through analysing a comprehensive whole slide image.

#### Sufficiency of selecting 30 tiles for each case

We performed a comprehensive analysis to the minimally necessary size of the candidate tile set selected from the tile classifications of the base classifier on the original whole slide image having the highest likelihood. We determined this number (CANDSIZE) by changing the number of top tiles fed to the ensemble models, ranging from just a single tile up to the top 200 on the classifier’s sensitivity on the subset of 100 slides from the training set. We explored different combinations of likelihood and cutoff values for classifying a slide as dual-stain positive. Our comprehensive analysis was conducted under three different STDMAX standard deviation settings (10, 15, 20). The analysis was performed on a subset of 100 slides from the training set. We selected 25 slides of each “CIN3+” and “<CIN2” from both the “Kaiser pilot” (SurePath^™^) and the “Biopsy” (ThinPrep^™^) studies. Using more than 10 tiles did not improve the classifier’s overall sensitivity (figure 7). The initial classification by the base classifier already detects dual-stain positive image tiles sufficiently well within the top 10. However, when the parameter setting included a 40% likelihood and a cutoff of 2, a decline in sensitivity was noted when fewer than 10 tiles were utilized for all standard deviations. For all other parameter combinations, a decrease in sensitivity was observed only when less than 3 tiles were considered. From this we concluded that 10 tiles selected with the base-model provides sufficient sensitivity for a whole dual-stain slide. To ensure a substantial safety margin (3x) for the detection of dual stain positive events, while also taking computational resource limitations into account, we selected a configuration with CANDSIZE = 30 tiles.

#### Ensembles stabilize likelihood predictions and narrow likelihood distributions

**Figure 3:**
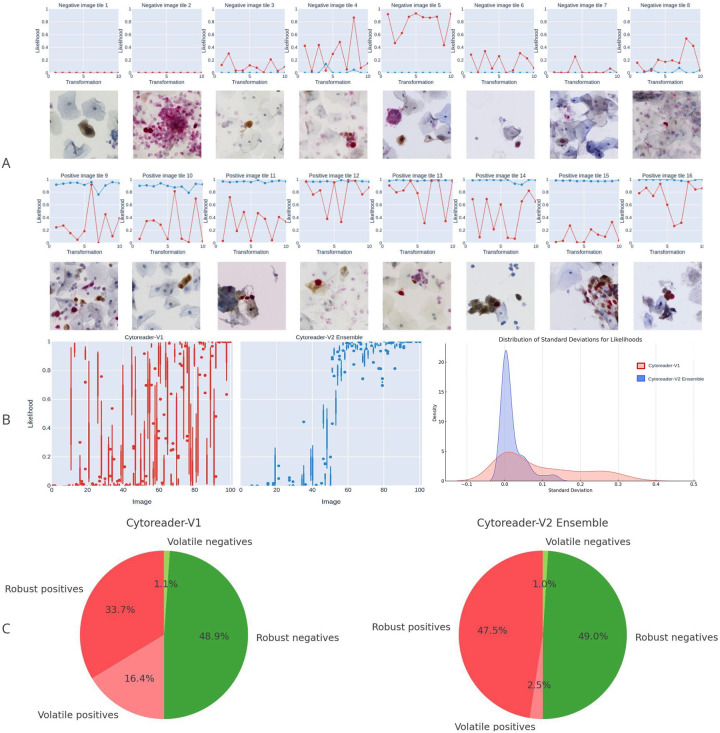
Increase in classification robustness through ensembles. (A) 100 Exemplary representative dual-stain negative and positive images from the curated dataset are presented. The first row displays 8 plots of the likelihoods alongside the corresponding negative image tiles in the second row. The third row shows 8 plots for the positive images in row four. The red lines represent the results from the Cytoreader-V1 model, and the blue lines depict the Cytoreader-V2 ensemble likelihoods. (B) Box plots of likelihood range distribution for the exemplary 100 images, with the Cytoreader-V2 ensemble displaying a narrower range (average 7.4%) compared to the Cytoreader-V1 model (average 28.0%). Density plot showing the reduced standard deviation of prediction likelihoods of ensembles, indicating more consistent predictions across transformations. (C) Increase of classification robustness through ensembles on 11,086 dual-stain positive and negative image tiles, not used for training, against Cytoreader V1. The ensemble shows more consistent curves, highlighting its stability, while Cytoreader-V1 exhibits greater variability along the transformations.

To assess tile level robustness we simulated different image transformations and analyzed the effects on a model by comparing the model predictions for the applied transformations 3-A. A highly robust model should exhibit minimal perturbations in response to such transformations, with predictions confined within a narrow probability range. We investigated the robustness of the ensemble model introduced in this study and compare it to our previously utilized model, the Cytoreader-V1 network. For this purpose, we selected a subset comprising 50 dual-stain negative and 50 dual-stain positive images from our training dataset not used for training. These images underwent systematic alterations through nine distinct image transformations: rotations (at 90, 180, and 270 degrees), horizontal and vertical flips, minor brightness adjustments (both increased and decreased), image blurring, and sharpening creating a set of 1,000 images (50 × 10 ds- & 50 × 10 ds+). Figure 3-A illustrates 8 exemplary dual-stain negative and, concurrently, positive images from the curated set. The likelihood values of the predictions across the original image and the 9 transformations are plotted. The Cytoreader-V2 ensemble model demonstrated more consistent bar plots, highlighting its stability. In comparison, the Cytoreader-V1 network showed greater variability, with a wider range of likelihoods in these images. On average, the Cytoreader-V2 ensemble exhibited a narrower likelihood distribution range of 7.43%. Conversely, the Cytoreader-V1 model resulted in a likelihood range of 28% on the tested samples. This broader range indicates greater variability in the Cytoreader-V1 model’s predictions across transformations for the same image. Figure 3-B shows the range distribution as box plots of 100 images of the Cytoreader-V2 ensemble compared to the Cytoreader-V1 network. This visualization highlights the increased stability of the ensemble compared to the prior single V1 network. Lastly, we used a density plot to study the standard deviation of predictions, as shown in Figure 3-B. The Cytoreader-V2 ensemble model, shown in blue, has a peak at a lower standard deviation, indicating its ability to give consistent predictions across transformations. Conversely, the Cytoreader-V1 model, in red, has a broader distribution, suggesting more variability in its predictions. This confirmed that the ensemble model is better at handling image transformations than the prior single network.

#### Gain in robustness through ensembles

We then systematically characterized robustness as depicted in Figure 3-C by analyzing robustness on 11,086 tiles of the original candidate image tile set not used for training. Specifically, 10 different image transformations were applied to each tile (*n*_ds+_ = 3339; *n*_ds-_ = 7747), and all augmented tiles were classified using the Cytoreader-V2 ensemble model. For comparison, the performance of the Cytoreader-V1 model was also assessed to determine the improvement through the ensemble. Robustness was calculated with a given likelihood threshold *θ* as:

$$\text{Robustness}(\%)=\frac{\#\text{Predictions}>\theta }{\text{Total}\;\text{Predictions}}\times 100$$

For instance, if an image from the positive set is classified consistently across all 10 transformations as positive, it achieves a robustness of 100%. If a tile did not show the same positive or negative result after the above perturbations it was rated “volatile”, otherwise “robust”. This analysis shows that compared to the base classifier, the ensemble particularly improves the positive tiles and reduces the fraction of volatile tiles to below 4%. The ensembles are therefore a great contribution to increasing classification robustness. The average robustness/volatility results for each validation set class are shown in Table 1.

The Cytoreader-V2 model achieved significantly higher robustness for the positive class (*ds+*) compared to V1, while both models exhibited similar robustness for the negative class (*ds−*). Cytoreader-V2 demonstrates remarkable robustness, achieving 95% robust positives, compared to only 67.3% (robust positives 33.7% + volatile positives 16.4%) for V1. This indicates that the V2 model is far better at consistently classifying positive tiles. Conversely, volatile positives, where predictions for positive tiles were inconsistent, were 5% for Cytoreader-V2 and a much higher 32.7% for V1, further underscoring the superior stability of the V2 model for positive tiles. For negative tiles, both models performed similarly well, with V2 achieving 97.7% robust negatives and V1 achieving 97.8%. Volatile negatives were minimal for both models, at 2% for V2 and 2.2% for V1, demonstrating comparable reliability in handling negative tiles. Overall, the data suggest that while both models handle negatives with high consistency, Cytoreader-V2 substantially outperforms Cytoreader-V1 in maintaining consistent predictions for positive tiles.

### 2.3 Externally assessed performance using blinded study data

We then set out to determine the performance of Cytoreader-V2 for dual-stain detection and study the influence of slightly different parameter configurations. Cytoreader-V2 was externally and independently validated on 3,803 patients of three different studies. External means hear that evaluation was performed by an independent, organizationally separated team of the US-NCI data, totally separated from the classifier development team which was blinded for any outcome on tile or slide level. Performance was measured by that external team against the histological ground truth which were also allowed to directly compare performance of Cytoreader-V2 against manual evaluation as well as the ASCUS+ PAP cytology which also were evaluated against ground truth. Receiver operator characteristics (ROC) curve analysis was conducted by comparing the number of DS-positive cells to the primary endpoints. The endpoint for all three methods (Cytoreader-V2, manual, PAP) was the detection of CIN3+ (cervical intraepithelial neoplasia grade 3 or worse).

#### Externally assessed performance in the Kaiser Implementation Study

**Figure 4:**
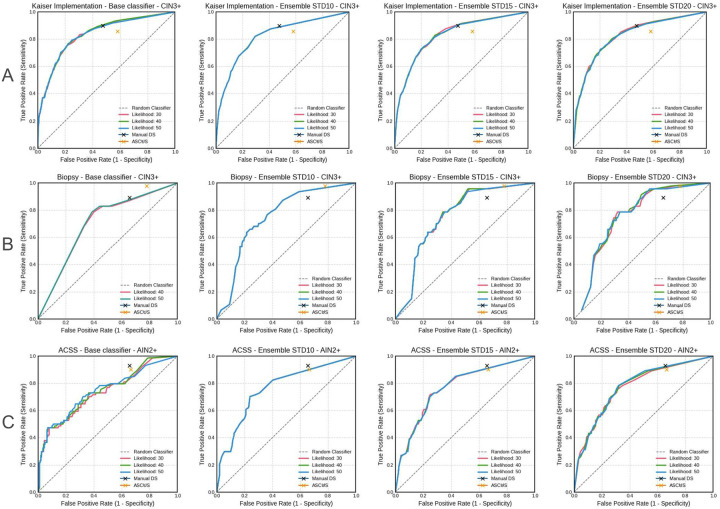
ROC curves in blinded external, blinded evaluation. Receiver operating curve characteristics analysis of the number of dual-stain (DS)-positive cells detected by the base classifier and the ensemble for detection of cervical precancer in three blinded epidemiological studies Kaiser Implementation (SurePath), Biopsy (ThinPrep) and ACSS (ThinPrep anal cancer) with 3,803 patients evaluated against histological ground truth (CIN3+ /AIN2+). Performance of STD10, STD15 and STD20 only differs marginally. For comparison manual DS reading and ASCUS+ cytology performance are marked in each graph. Base classifiers always deliver all top 30 tiles resulting in a sensitivity above 94% (see dedicated analysis) and are only shown for comparison here. Blue arrows indicate maximal specificity of Cytoreader V2 within acceptable sensitivity. Given deltas Δ*SP*_PAP_ and Δ*SP*_*M*_ mark the maximal gain in specificity of automatic dual-stain reading against ASCUS+ and manual dual-stain reading.

In this external validation using the ***Kaiser Implementation Study*** (n = 3095, with 218 cases of CIN3+), as an example of an HPV screening population, manual DS+ (serving here as a reference for the automatic classification) exhibited a sensitivity of 89.9% and a specificity of 52.6%. Table 2 provides a comparison of the results across the relevant parameters (see Classification Workflow). Figure 4-A displays the corresponding ROC curves. All ensembles generally maintained comparable sensitivity and specificity compared to manual DS. Optimal results in the Kaiser Implementation Study were achieved with the parameters STD20 ensemble at a cutoff of 2 DS-positive cells and a LH=50% likelihood which provides a sufficient sensitivity (87.2% vs. 89.9% manual and 85.8% PAP cytology) and superior specificity (57.8% vs. 52.6% manual and 41.9% PAP cytology).

#### Externally assessed performance in the Biopsy Study

In this external validation using the ***Biopsy Study*,** which is a colposcopy referral population (n = 409 patients with 53 cases of CIN3+), manual DS+ (serving here as a reference for the automatic classification) demonstrated a high sensitivity of 89.4% and a lower specificity of 34.6%, reflecting that this is a referral population in which cases have been referred to colposcopy from manual positive cytology reads. Table 2 presents comparisons across the relevant parameters with the corresponding ROC curves shown in Figure 4-B. The ensemble shows higher specificity compared to the base classifier while maintaining high sensitivity. The ensemble’s sensitivity is apparently higher than the base classifier’s as the base classifier is only used to generate a tile candidate set for the ensembles. Identical as in the Kaiser Implementation Study, in the Biopsy Study best results were achieved with the parameters STDMAX=STD20, CUTOFF=2 DS-positive cells and a LH=50% likelihood which provides a sufficient sensitivity (95.7% vs. 97.8% manual) and superior specificity (44.4% vs. 34.6% manual).

#### Externally assessed performance in the ACSS Study

To test generalization of the classifier, we then externally assessed classification performance in anal cancer screening in the ***ACSS Study*** (n=299 patients comprising 69 cases of AIN2+) which is also a referral population. We emphasize that the ACSS Study, as an example of positive samples selected in primary cytology screening for anal cancer, in an anoscopy population, was not included in the training set and therefore served here only as an informative analysis to explore a potential use in anal cancer. Manual DS+ (serving here as a reference for the automatic classification) demonstrated a sensitivity of 93.2% and a specificity of 34.4%. Table 2 presents comparisons across the relevant parameter while the corresponding ROC curves are shown in Figure 4-C. The ensemble methods generally showed slightly reduced sensitivity but improved specificity compared to the base classifier. Specifically, the STD20 ensemble at a cutoff of 1 DS-positive cell and a 50% likelihood achieved a sensitivity of 89.2% and a specificity of 49.3%, with an AUC of 0.78 and thus comparable sensitivity and superior specificity when compared to manual DS.

#### Summary of results

Externally and independently assessed blinded performance results from the three studies Kaiser Implementation Study (SurePath), the Biopsy Study (ThinPrep) and ACSS (ThinPrep) showed equal sensitivity and superior specificity compared to manual DS and cytology as standard of care. For cervical cancer screening we obtained a standardized parameter configuration of LH=50% and STDMAX=20% and cutoff of 2 and for anal cancer, a cutoff of 1.

## 3 Discussion

The p16/Ki67 dual stain has shown great promise for triage of HPV-positive individuals in cervical cancer screening. Widespread introduction and adoption in clinical laboratories will be facilitated through automated DS algorithms. Automation can serve as a scalable, quality control approach when introducing and evaluating the assay and subsequently be used for semi-assisted or fully automatic assay interpretation. Cytoreader V2 demonstrated high diagnostic performance across multiple datasets while maintaining manageable technical and regulatory complexity. We show that the trained single tile classifiers nearly have perfect accuracy. So increasing the complexity of the approach may not necessarily enhance classification accuracy but risk overfitting instead in overly broad models with numerous parameters [40]. Our development process shows that accuracy of tile recognition is sufficiently high, thus increasing complexity cannot be expected to increase slide-level performance much further. All steps in the pipeline are understandable and have specific and interpretable functions like generating tile candidates by the base classifier, enhancing robustness of the classifications (by test time augmentation and ensembles) and filtering for ambiguity. This transparent and quantifiable approach supports regulatory evaluation and reduces the risk of overfitting by using overly complex and parameter-rich deep learning methods. These aspects underscore the practicality of our approach and explainability supporting medical acceptance and regulatory approval in medical AI which is inherently adaptable to other AI applications in digital pathology.

Cervical cancer screening is one of the most widely implemented and successful approaches to cancer prevention and is currently undergoing substantial evolution, transitioning away from conventional cytological PAP screening. Automating the dual-stain test with AI addresses a critical gap in cervical cancer screening, and can make high quality triage of HPV-positives available in underserved regions. Cytoreader-V2 provides consistent sensitivity and increased specificity, reduces human workload, and enables broader access to life-saving diagnostics. Given the global burden of cervical cancer, this innovation represents a paradigm shift in the application of digital pathology for women’s health.

### Overall performance

We have characterized the performance of Cytoreader-V2 as comparable in sensitivity and superior in specificity in an externally validated, blinded evaluation, although its semi-automatic, assisted use is also an option. We externally had the performance of our AI setup evalated relative to histological ground truth, so that AI and human DS reading as well as ASCUS+ cytology as the standard of care, can be compared directly. This provides an exceptional degree of validation for computational AI in a clinically realistic setting. As outlined in the recently modified US-guidelines for cervical cancer screening, dual-stain reading will be deployed practically as a triage step subsequent to HPV screening enhancing its specificity [2, 3, 4]. Therefore, specificity is an important parameter in our objective performance in its comparison against manual dual-stain reading. Nevertheless, sensitivity against ground truth must be kept sufficiently high according to current diagnostic standards to not miss cases. Our model training and internal validation shows sufficiently high accuracy on the tile- and slide level. The stark difference between the near perfectly classifying tile-level classification with an accuracy above 96% and the slide-level diagnostics against ground truth is typical of real-world AI applications although still beating human manual dual-stain reading. This discrepancy between the very high sensitivity and specificity observed at the tile level and the somewhat lower performance against clinical outcome, represents a typical, yet unavoidable challenge in realistic manual and automated diagnostics. Therefore, if available, the use of a shared ground truth is important in diagnostic studies.

### Analysis of robustness

One of the critical achievements of Cytoreader-V2 is its introduction of a second confidence parameter next to likelihood for each tile prediction to achieve an enhanced overall robustness. As demonstrated in our robustness analysis, the combined test-time augmentation ensemble model is significantly less affected by image transformations, staining variability, and slide preparation differences than its predecessor, Cytoreader-V1. The ensemble model’s stability across diverse image modifications, as evidenced by a narrower range of predictive likelihoods, confirms that it is suited for real-world applications where such variations are inevitable.

### Cross-comparison on study performances

Cytoreader-V2 can accurately identify true positives (CIN3+ cases) while reducing the number of false positives. The observed elevated specificity while also having using one unified classifier for ThinPrep and SurePath is important. Cytoreader-V2 has the ability to reduce false positives, thereby minimizing unnecessary colposcopies and biopsies, therefore reducing the burden of follow-up procedures in cervical cancer screening. Generally we would like to point out that Cytoreader-V2 was compared to highly trained experts and a comparison against less skilled readers can thus be expected to yield a stronger performance difference. The benefit of AI-based dual-stain reading will especially lie within its capability for widespread and rapid implementation of the dual-stain assay also in labs with less routine experience with this assay. Furthermore, as in [13, 39] we further confirm Cytoreader’s potential for anal cancer screening. This cross-applicability indicates the robustness of the AI platform and highlights its potential for broader clinical utility beyond cervical cancer. The improved robustness and accuracy also hold promise for low-resource settings where access to expert pathologists and high-quality infrastructure is limited. Automated DS evaluation paired with digital slide scanning allows for remote processing and analysis, meaning that samples can be scanned in one location and analyzed in another. This geographic flexibility could significantly improve screening capabilities in under-served areas, where cervical cancer rates remain high.

### Summary

This study demonstrates that automating the dual-stain test with AI has the potential to transform cervical cancer screening globally. Cytoreader-V2 improves diagnostic accuracy and robustness, achieving high sensitivity and specificity while addressing real-world variabilities. The clearly explainable model classification parameters support key regulatory requirements of robustness, interpretability, and scalability. Nevertheless, more studies for validating Cytoreader are needed and underway. Medically, it facilitates real-world implementation and can reduce the burden on healthcare systems. Its adaptability extends beyond cervical cancer screening, establishing a foundation for broader applications of AI in digital pathology and cancer screening in general.

## 4 Methods

### 4.1 Study populations

Based on image data from the Kaiser Permanente Norther California (KPNC) Dual Stain Implementation Study [36], the NCI Biopsy Study [8, 14], the Improving Risk Informed HPV Screening study IRIS [17, 32], the study STRIDES Studying Risk to Improve DisparitiES in Cervical Cancer in Mississippi [16, 32], and ACSS, the Anal Cancer Screening Study [15, 39], we developed and evaluated Cytoreader-V2. Cytoreader-V2 is building on our earlier work [13]. We selected 5,722 whole slides of which 1,919 were used for training and 3,803 were designated as the test set for validation. All slides were prepared using either ThinPrep^™^ or SurePath^™^ methods. The slides were scanned at 20× magnification to produce high-resolution Whole Slide Images (WSIs) using the Hamamatsu Nanozoomer S360 or Nanozoomer HT scanner.

### 4.2 Clinical endpoints

All studies followed routine clinical practice at their respective medical institutions. Cytological evaluations were categorized in accordance with the Bethesda System, encompassing negative findings for intraepithelial lesions or malignancy, atypical squamous cells of undetermined significance, low-grade squamous intraepithelial lesions, and high-grade squamous intraepithelial lesions (HSIL) [28]. The definitive diagnosis, used by us as “ground truth”, was determined through histopathological assessments, employing the cervical intraepithelial neoplasia (CIN) terminology for cervical outcomes. This scale delineates the degree of dysplasia in the cervical epithelium, ranging from normal to CIN, grade 1 (CIN1), grade 2 (CIN2), grade 3 (CIN3), and cancer. Adenocarcinoma in situ was grouped with CIN3. Anal disease endpoints were categorized using the analogous anal intraepithelial neoplasia nomenclature (AIN).

### 4.3 Generation of model training slide and tile set

**Figure 6:**
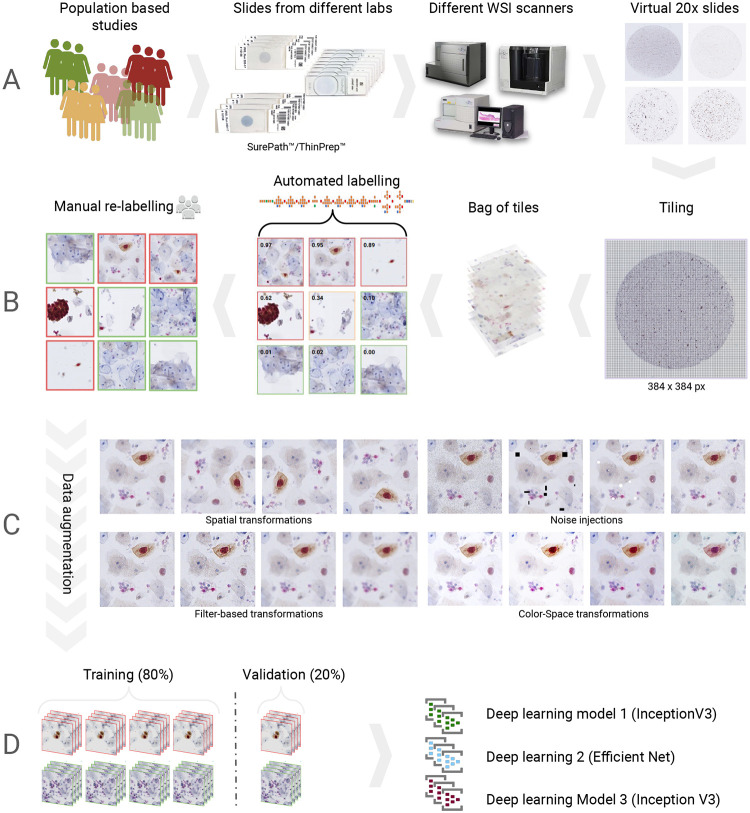
Workflow for training set generation and model training. (A) Sample Acquisition: Utilization of specimens from diverse population-based studies. Application of slide preparation techniques including ThinPrep^™^ and SurePath^™^. Slides undergo scanning at 20× magnification, yielding high-resolution Whole Slide Images (WSIs). (B) Image Tile Extraction and Annotation: Tiles are derived from the WSIs and are subjected to automated labeling by the Cytoreader Network followed by manual re-labeling. (C) Data Augmentation: Rigorous data augmentation techniques are employed, encompassing spatial transformations, color space modifications, noise injections, and filter-based transformations. D) Neural Network Training: Post-augmentation images are partitioned into training and validation sets. These sets facilitate the training of multiple deep neural networks.

**Figure 7:**
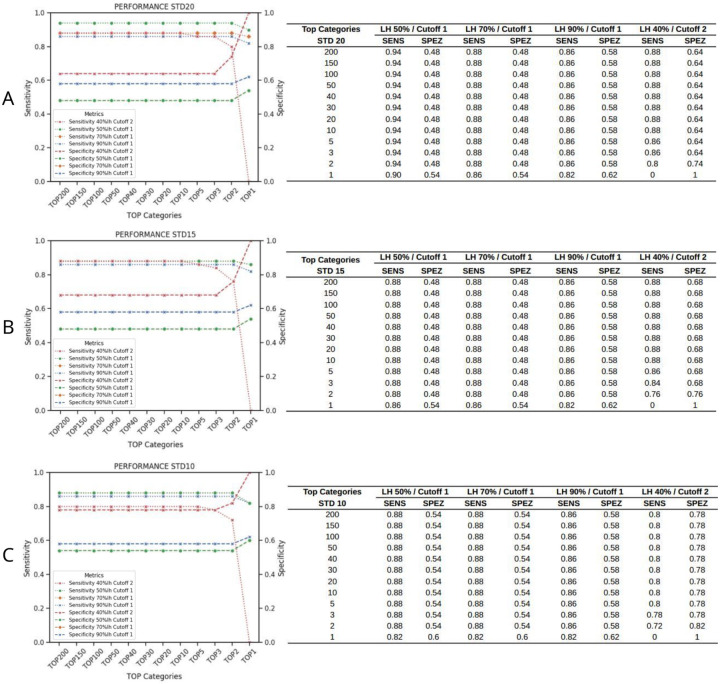
Determining that 10 top tiles are sufficient for dual-stain detection. (A) A standard deviation (STD) of 20 yields the highest sensitivity observed in the training set. Applying a cutoff of 2 in conjunction with a 40% likelihood combination and using fewer than 10 tiles results in reduced sensitivity. (B) Utilizing a reduced STD of 15 yields an overall diminished sensitivity, yet maintains the same specificity. There is an observed sensitivity reduction when fewer than 3 tiles are used for a cutoff of 1, and when less than 10 tiles are used for a cutoff of 3. (C) Adopting a STD of 10 enhances specificity across all parameter configurations. The decline in sensitivity mirrors the patterns observed under A and B.

We first assembled a training slide set of 1,919 dual-stain positive and negative training slides from which a base network as an initial classifier as well as ensemble networks are trained. As we aimed for a unified classifier to work with comparable performance on ThinPrep and SurePath slides, this training slide set comprised example slides of both liquid cytology preparation methods. All slides were stained with the CINtec Plus dual-stain assay. Training of all neural networks was performed on 384×384 pixel tiles extracted from this dual-stain training slide set. In order to streamline training set development, we used the Cytoreader-V1 [13] network to select additional tile candidates for our training set enlarging the previously used tile set. For optimal separation of positive and negative tiles we extracted tiles with specific likelihood ranges as listed in Table 2. This allowed us to deliberately create a tile candidate set with enhanced performance in the classification “gray zone”. We then subjected this tile candidate set to manual expert review to create the full training tile set. This way, we created a likelihood-based iterative refinement approach for automatic pre-labelling with subsequent final expert labelling in order to enhance the precision of the trained classifier. With this semi-automatic approach, we increased our dataset from 20,625 to 55,430 image tiles (384×384 pixels), a resolution large enough to capture neighboring cells and larger cell clusters, see Table 3. All “green” tiles in the respective likelihood range were manually labelled as negative, while “red” tiles were selected as positive. Also a sample of 1,000 automatically labeled negative tiles that was manually checked, revealed a true negative rate of 99.9% which meant that we could not sample overlooked tiles using Cytoreader-V1 pre-selection. The resulting full training set was then split into 80% for training and 20% for validation. Specifically, the training subset comprised 16,500 ds+ image tiles and 38,930 ds- image tiles, while the validation subset consisted of 3,339 ds+ and 7,747 ds- image tiles. The overall iterative training set generation procedure is depicted in detail in Figure 6 A–B.

### 4.4 Training of the base classifier and ensemble

Two variants of the InceptionV3 [30] architecture (labeled as *IncV*3_1_ and *IncV*3_2_) and one instance of the EfficientNetV2B2 [26] (*EffNet*_1_) were utilized for training. We adapted the networks’ final classification layers to produce a two-class output. In our methodology, a robust test-time data augmentation strategy was implemented including rotation, flipping, brightness adjustments, color value adjustments, various blurring methods, edge transformations, and morphological transformations. A random selection process determined the application of the augmentations, ensuring variability in the augmented dataset. Additional modifications like color enhancement were conditionally applied further diversifying the training data.

## Figures and Tables

**Figure 5:**
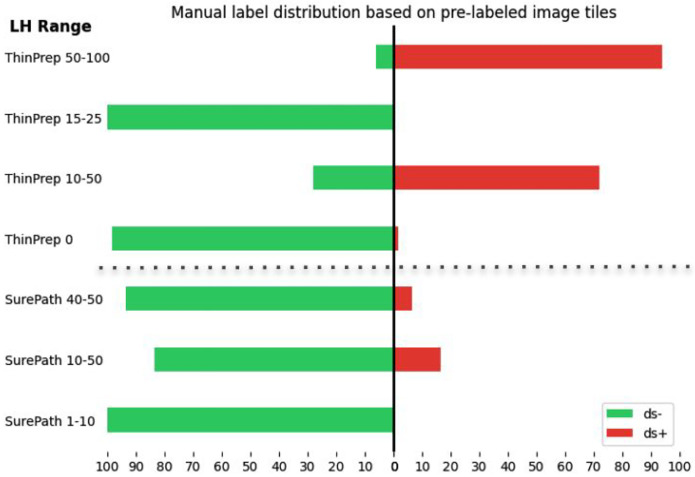
Distribution of manually labeled image tiles folowing pre-classification by the Cytoreader-V1 network. The chart illustrates the percentage of manual labels applied to pre-classified image tiles, categorized by their respective likelihood ranges. ThinPrep image tiles in the likelihood range of 10–50 were predominantly labeled as positive (ds+), whereas most tiles in the same range at SurePath tiles were labeled as negative (ds−), indicating the lower sensitivity for ThinPrep slides of the Cytoreader-V1 network.

**Table 1:** Average robustness results for the Cytoreader V1 and V2 models across validation set classes.

Network	Class	Robustness (%)
Cytoreader-V1	ds+	67.3
Cytoreader-V1	ds−	97.8
Cytoreader-V2	ds+	95.0
Cytoreader-V2	ds−	97.7

**Table 2:** Quantitative blinded, externally validated performance on multiple combinations of CUTOFF, Likelihood LH and STDMAX. Performance data for Implementation (CIN3+), Biopsy (CIN3+) and Anal Cancer Screening Study (AIN2+) are given. Automated vs. Manual Detection of DS-positive Cells in Various Studies.

Study	Kaiser Implementation (N = 3095)					Biopsy Study (N = 409)					Anal Cancer Screening (N = 299)				
Algorithm	Sens.	Spec.	YI	CU	LH	Sens.	Spec.	YI	CU	LH	Sens.	Spec.	YI	CU	LH
**Manual DS**	**89.9**	**52.6**	42.6	-	-	**89.4**	**34.6**	23.9	-	-	**93.2**	**34.4**	27.6	-	-
**ASCUS+ cytology**	**85.8**	**41.9**	27.6	-	-	**97.8**	**22.3**	20.1	-	-	**90.4**	**33.5**	27.6	-	-
Base Classifier	94.0	39.4	33.5	1	30	-	-	-	1	30	98.7	17.9	16.6	1	30
Base Classifier	89.9	53.9	43.8	2	30	-	-	-	2	30	91.9	25.9	17.8	2	30
Base Classifier	93.1	42.4	35.5	1	40	87.2	34.1	21.4	1	40	98.7	21.4	20.0	1	40
Base Classifier	89.9	56.1	46.0	2	40	83.0	46.8	29.8	2	40	89.2	29.4	18.5	2	40
Base Classifier	91.7	44.9	36.7	1	50	87.2	35.8	23.0	1	50	93.2	22.9	16.1	1	50
Base Classifier	87.6	58.3	45.9	2	50	83.0	48.8	31.8	2	50	85.1	30.9	16.0	2	50
Ensemble STD10	87.6	58.7	46.3	1	30	93.6	40.6	34.2	1	30	82.4	59.7	42.1	1	30
Ensemble STD10	82.1	70.4	52.6	2	30	87.2	52.6	39.8	2	30	73.0	69.2	42.1	2	30
Ensemble STD10	87.6	58.8	46.4	1	40	93.6	41.0	34.6	1	40	82.4	59.7	42.1	1	40
Ensemble STD10	82.1	70.5	52.6	2	40	87.2	52.6	39.8	2	40	73.0	69.2	42.1	2	40
Ensemble STD10	87.6	58.8	46.4	1	50	93.6	41.0	34.6	1	50	82.4	59.7	42.1	1	50
Ensemble STD10	82.1	70.5	52.6	2	50	87.2	52.6	39.8	2	50	73.0	69.2	42.1	2	50
Ensemble STD15	91.7	49.3	41.1	1	30	95.7	33.2	28.9	1	30	85.1	55.7	40.9	1	30
Ensemble STD15	87.6	61.8	49.4	2	30	95.7	47.1	42.8	2	30	77.0	65.2	42.2	2	30
Ensemble STD15	91.3	50.7	42.0	1	40	95.7	35.2	30.9	1	40	85.1	56.7	41.9	1	40
Ensemble STD15	84.9	63.2	48.1	2	40	95.7	48.1	43.9	2	40	77.0	65.2	42.2	2	40
Ensemble STD15	90.8	51.4	42.2	1	50	95.7	35.5	31.2	1	50	85.1	56.7	41.9	1	50
Ensemble STD15	84.9	63.9	48.8	2	50	93.6	48.8	42.4	2	50	77.0	65.2	42.2	2	50
Ensemble STD20	93.1	39.1	32.2	1	30	97.9	25.3	23.1	1	30	89.2	44.3	33.5	1	30
Ensemble STD20	90.4	52.0	42.3	2	30	95.7	35.8	31.6	2	30	82.4	56.7	39.2	2	30
Ensemble STD20	92.2	42.6	34.8	1	40	97.9	28.7	26.5	1	40	89.2	46.8	36.0	1	40
Ensemble STD20	88.5	54.8	42.3	2	40	95.7	40.6	36.4	2	40	82.4	60.2	42.6	2	40
Ensemble STD20	91.3	45.3	36.6	1	50	95.7	32.1	27.8	1	50	**89.2**	**49.2**	38.4	1	50
**Ensemble STD20**	**87.2**	**57.8**	44.9	2	50	**95.7**	**44.4**	40.11	2	50	82.4	61.2	43.6	2	50

YI = Youden’s Index; CU = Cutoff; LH = Likelihood Threshold.

**Table 3:** Composition of the training set. Tiles were categorized by preparation type, labeling method, and likelihood (LH) range. The tiles are sourced from SurePath and ThinPrep slides, with final labeling performed manually and some preliminary labelling from the Cytoreader-V1 network. The table includes counts for dual-stain positive (ds+) and dual-stain negative (ds−) tiles across various LH ranges. The total counts for the entire dataset, as well as the specific breakdown between the training set (80%) and validation set (20%), are provided, highlighting a total of 16,500 ds+ tiles and 38,930 ds- tiles used for model training and validation.

Type	Labeling	LH range	ds+	ds−	Total
SurePath	Cytoreader + Manual	1–10	0	3,705	3,705
SurePath	Cytoreader + Manual	40–50	189	2,688	2,877
SurePath	Cytoreader + Manual	10–50	938	4,807	5,745
ThinPrep	Cytoreader + Manual	10–50	2,943	1,153	4,096
ThinPrep	Cytoreader + Manual	15–25	3	3,624	3,627
ThinPrep	Cytoreader + Manual	50–100	2,837	188	3,025
ThinPrep	Cytoreader + Manual	/	190	11,540	11,730
SurePath/ThinPrep	Manual	/	9,400	11,225	20,625
		Total	16,500	38,930	55,430
		Trainset 80%	13,355	30,989	44,344
		Valset 20%	3,339	7,747	11,086

## Data Availability

De-identified study data used in this article will be made available and shared for research purposes in accordance with institutional review boards and according to NCI data sharing principles on request to N.W. (wentzenn@mail.nih.gov). Images of dual-stain tiles and their labels from the validation dataset will be shared upon written request to academic investigators without relevant conflicts of interest for non-commercial use who agree not to distribute the data. Access requests can be made to N.G. (niels.grabe@stcmed.com).
